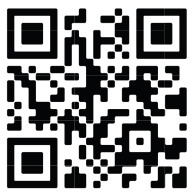# Tubal flushing with oil-based contrast during transvaginal hydro laparoscopy, a case report 

**DOI:** 10.52054/FVVO.14.2.019

**Published:** 2022-07-01

**Authors:** I Roest, A.M. Hajiyavand, K.D. Dearn, M.Y. Bongers, V Mijatovic, B.W.J. Mol, C.A.M. Koks

**Affiliations:** Department of Obstetrics and Gynaecology, Máxima MC, Veldhoven/Eindhoven, the Netherlands; Department of Reproductive Medicine, Amsterdam UMC, Vrije Universiteit Amsterdam, Amsterdam Reproduction and Development, Amsterdam, the Netherlands; Grow research school for Oncology and Reproduction, Maastricht University, Maastricht, The Netherlands; Mechanical Innovation and Tribology Group, Department of Mechanical Engineering, School of Engineering, University of Birmingham, Birmingham, B15 2TT, UK; Department of Obstetrics and Gynaecology, University of Monash, Melbourne, Australia

**Keywords:** Case report, Feasibility, Fertility, Oil-based contrast, Transvaginal hydro-laparoscopy

## Abstract

**Background:**

Oil-based contrast has been shown to have a fertility-enhancing effect during hysterosalpingography (HSG) but is not yet used during transvaginal hydro laparoscopy (THL).

**Objective:**

To asses if additional tubal flushing with oil-based contrast during THL is feasible.

**Materials and Methods:**

Case report with video assessment. A healthy 29-year-old woman with primary unexplained subfertility, underwent a THL under local anaesthesia. First, chromopertubation was performed by methylene blue. Afterwards, tubal flushing with 3mL oil-based contrast (Lipiodol® UltraFluid, Guerbet) was performed.

**Main outcome measures:**

In this case report we evaluated the feasibility of additional tubal flushing with oil- based contrast during THL, in terms of; the visibility of the oil-based contrast at the tubal fimbriae, the pain and acceptability scores.

**Results:**

Both fallopian tubes were patent to methylene-blue as well as to oil-based contrast. Interestingly, the oil-based contrast came out of the fallopian tube in the form of free droplets with strong internal bonding. Furthermore, some residue of the droplets was visible on the surface of the peritoneal wall in the form of oily micro-droplets.

**Conclusions:**

We present the first sub-fertile woman, in which additional tubal flushing with oil-based contrast during THL was performed. It is likely, that the residue of oily micro-droplets is also present inside the fallopian tube, where it may enhance the cilia movement by introducing lubrication. These lubricating characteristics of the oil-based contrast may be important for its fertility-enhancing effect. More research is necessary to confirm this hypothesis and the feasibility of tubal flushing with oil-based contrast during THL in more women.

## Learning objective

Oil-based contrast is frequently used during hysterosalpingography (HSG) in subfertile women, because of its fertility enhancing effect. Transvaginal hydro laparoscopy (THL) is an alternative procedure, which explores the tubo- ovarian structures and the pouch of Douglas, in addition to tubal patency. However, so far, only water-based media are used during THL. The aim of this case report was to determine if additional tubal flushing with oil-based contrast was feasible during THL. We observed that oil-based contrast forms micro-droplets which may enhance the tubal cilia movement due to lubrication, this is one of the hypotheses for the fertility enhancing effect of oil-based contrast.

## Introduction

Subfertility is defined as the lack of conception after 12 months of timed unprotected intercourse (Zegers-Hochschild et al., 2009). Unfortunately, around 15% of couples suffer from subfertility (Thoma et al., 2013). During the fertility work-up in these couples, tubal patency testing, traditionally in the form of hysterosalpingography (HSG), is often performed. A previous study showed that a HSG with an oil-based contrast has a positive effect on the natural conception rate, compared to a HSG with water-based contrast (van Rijswijk et al., 2020). Transvaginal hydro laparoscopy (THL) is an alternative procedure to test tubal patency. The benefit of THL is the possibility to explore the tubo- ovarian structures and the pouch of Douglas, neither evaluated during a HSG (Gordts et al., 1998; Gordts et al., 2021). However, during a THL procedure, a water-based medium (methylene blue) is used instead of an oil-based medium, of which no fertility enhancing effect has been shown.

This case report is the first direct observation of oil- based contrast spill from the tubal fimbriae in-vivo.

## Patient and method

A 29-year-old woman (nullipara) was referred to the fertility department of our hospital, because of primary subfertility with her current partner for almost 3 years. She had no history of sexually transmitted diseases or abdominal surgery. Aside from prenatal multivitamins, including folic acid and vitamin D, she did not take any medication. Her menstrual cycle was regular at an interval of 30 days. At presentation, she smoked around ten cigarettes per day. Her body mass index was 26.7 kg/m^2^. Transvaginal ultrasonography did not show any abnormalities of the uterus and both ovaries had a multifollicular aspect. Blood examination was negative for chlamydia antibody titre and showed a normal thyroid-stimulating hormone (TSH) level (1.7 m[IU]/L). The semen analysis of her partner showed 240 million motile spermatozoa.

Because of prolonged subfertility, tubal testing was performed. The woman underwent a THL, which is the first line tubal testing method at our clinic. The THL procedure was performed at the outpatient department with local anaesthesia, as is standard at our clinic for both THL and HSG, using the reusable trocar system (Storz), by a specialized gynaecologist (CK). Details of the procedure have been previously described (Coenders-Tros et al., 2016). First, chromopertubation was performed by the use of 6mL methylene blue. After the diagnosis of tubal patency, an additional tubal flushing with 3mL oil-based contrast (Lipiodol ® Ultra Fluid, Guerbet Netherlands, Gorinchem) was performed. The infusion of the contrast was stopped when spill from both fallopian tubes had been observed, in this case the amount of contrast used was relatively low.

## Results

**Figure 1:**
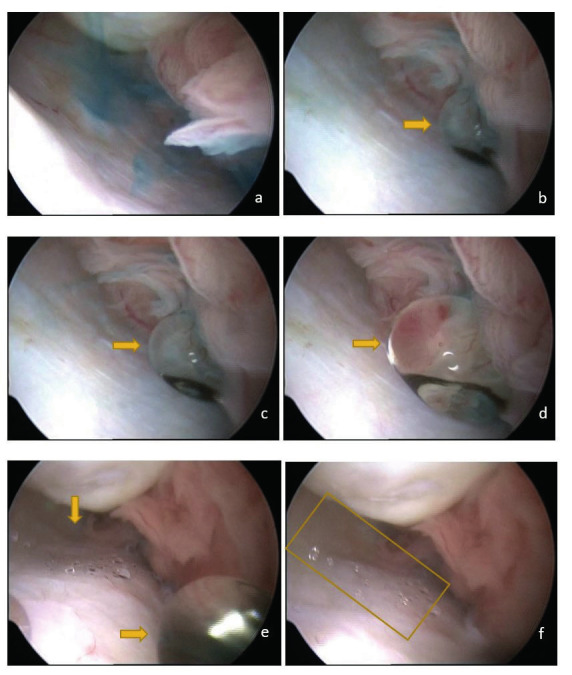
a) Dispersion of methylene-blue from the tubal fimbriae. b, c, d) Oil-based contrast exits the tubal fimbriae as a droplet. e, f) A trace of oil-based contrast in the form of micro-droplets is left on the peritoneal wall.

Inspection at the start of the THL procedure showed no abnormalities of the fallopian tubes or the abdominal cavity. The video (see the QR-code) starts when the camera is positioned at the fimbrial end of the fallopian tube. The abdominal cavity is infused with pre-warmed saline water (NaCl 0.9%, 37 °C). As soon as the methylene-blue exits the fallopian tube, it immediately disperses in the saline solution, without forming a droplet (See Figure 1a). Afterwards, the oil-based contrast (Lipiodol® Ultra Fluid) comes out of the fallopian tube in the form of free droplets (See Figure 1b, c, d). When the first droplet moves out of the scope of the camera, almost directly a second droplet forms, some residue of the droplet of oil-based contrast is visible on the surface of the peritoneal wall in the form of micro-droplets (See Figure 1e, f). Additionally, Figure 1d shows that the droplet of oil-based contrast has strong internal bonding (surface tension) because it does not disperse when it comes in contact with the tubal fimbriae.

The pain score, reported on the Visual Analogue Scale by the patient, was 50 mm during the installation of methylene-blue and 70mm during the instillation of the oil-based contrast (0 mm no pain, 100 mm worst possible pain). The procedure, including the additional flushing with oil-based contrast, was moderately acceptable to the patient (5 out of 10, 10 being completely acceptable, 0 completely unacceptable). Furthermore, she would advise friends or family to undergo the same procedure (10 out of 10, 10 being the highest score, 0 the lowest). Blood examination four weeks after the procedure showed a TSH of 3.1 m[IU]/L. The couple was counselled for expectant management, with timed intercourse, for the next few months.

## Discussion

To the best of our knowledge, this is the first patient in which tubal flushing with oil-based contrast has been directly visualised during a THL-procedure. In this case, tubal flushing with an oil-based contrast during THL was feasible and acceptable to the patient; however, further studies need to be performed to confirm these observations.

The oil-based contrast used in this case, Lipiodol® Ultra Fluid, is a combination of iodine and fatty acid ethyl esters of poppyseed oil (Guerbet, Netherlands). The oil-based contrast is non-water soluble, hence it doesn’t mix with saline water, and it forms an emulsion in water. Due to the high density of this oil-based contrast compared to saline water (1.28 g/cm3 for Lipiodol® Ultra Fluid and 1.00 g/cm3 for 0.9% saline water at 37 °C), the oil-based contrast moves in the form of a droplet (See Figure 1b). The droplets of oil-based contrast have a high surface tension, therefore they have a strong internal bonding and the droplets do not break when they come into contact with the tubal fimbriae. Furthermore, the video as well as the images show that the droplets of oil-based contrast leave a trace (a residue) on the surfaces of the peritoneal wall once it moves away (See Figure 1 e f). This residue is in the form of micro-droplets, which is also referred to as the wetting properties of the oil-based contrast. It is likely that this sequence of events also happens inside the fallopian tube. The residue of oil-based contrast inside the fallopian tube and in between the cilia may enhance the cilia movement, by introducing lubrication in between the cilia of the tubal epithelium. This lubrication effect of oil-based contrast is one of the hypotheses for the fertility enhancing effect of tubal flushing (Roest et al., 2022). It is also possible to attempt salpingoscopy during a THL, which may visualise the aspect of the residue of oil-based contrast inside the fallopian tube (Suzuki et al., 2005). However, salpingoscopy was not performed in this case.

The main concern against the standard use of oil- based contrast in tubal patency testing, without fluoroscopic guidance, is the risk of intravasation of the contrast media and the 1subsequent risk of developing an oil-embolism (Roest et al., 2021). With the use of fluoroscopy guidance, the instillation of the contrast media can be directly halted if intravasation occurs, without fluoroscopy guidance intravasation may not be detected. Therefore, we propose to only use oil-based contrast during THL as an additional flushing medium, after the diagnosis of at least unilateral tubal patency by the use of water-based contrast. Additionally, we advise to use a limited amount of oil-based contrast, in this case 3mL was used. We propose the same restrictions when using oil-based contrast during laparoscopic tubal patency testing.

## Conclusions

Concluding, additional tubal flushing with oil- based contrast is possible during THL and probably laparoscopy, without the use of fluoroscopic guidance. However, additional research is needed to determine the feasibility and safety in more patients. Our research group has started a prospective cohort study to examine the feasibility of additional tubal flushing with an oil-based contrast during THL in 50 women. Furthermore, the characteristics of an oil-based contrast, especially the lubricating effect, may be the reason for the fertility enhancing effect of tubal flushing with oil-based contrast compared to water-based contrast. The direct observation of the oil-based contrast residue, micro-droplets, on the peritoneal wall is an important step in gaining more knowledge on the characteristics of oil-based contrast.

## Video scan (read QR)

https://vimeo.com/esge/review/665957218/0c3d5e917d

**Figure:**